# Cognitive Profiles of Aging in Multiple Sclerosis

**DOI:** 10.3389/fnagi.2019.00105

**Published:** 2019-05-10

**Authors:** Dejan Jakimovski, Bianca Weinstock-Guttman, Shumita Roy, Michael Jaworski, Laura Hancock, Alissa Nizinski, Pavitra Srinivasan, Tom A. Fuchs, Kinga Szigeti, Robert Zivadinov, Ralph H. B. Benedict

**Affiliations:** ^1^Department of Neurology, Jacobs School of Medicine and Biomedical Sciences, University at Buffalo – The State University of New York, Buffalo, NY, United States; ^2^Buffalo Neuroimaging Analysis Center, Department of Neurology, Jacobs School of Medicine and Biomedical Sciences, University at Buffalo – The State University of New York, Buffalo, NY, United States; ^3^Department of Neurology, University of Wisconsin School of Medicine and Public Health, Madison, WI, United States; ^4^Clinical Translational Science Institute, Center for Biomedical Imaging, University at Buffalo – The State University of New York, Buffalo, NY, United States

**Keywords:** aging, MS, cognition, processing speed, verbal fluency

## Abstract

**Background:**

Increasingly favorable mortality prognosis in multiple sclerosis (MS) raises questions regarding MS-specific cognitive aging and the presence of comorbidities such as Alzheimer’s disease (AD).

**Objective:**

To assess elderly with MS (EwMS) and age-matched healthy controls (HCs) using both MS- and AD-specific psychometrics.

**Methods:**

EwMS (*n* = 104) and 56 HCs were assessed on a broad spectrum of language, visual-spatial processing, memory, processing speed, and executive function tests. Using logistic regression analysis, we examined cognitive performance differences between the EwMS and HC groups. Cognitive impairment (CI) was defined using a -1.5 SD threshold relative to age and education years-matched HCs, in two cognitive domains.

**Results:**

CI was observed in 47.1% of EwMS with differences most often seen on tests emphasizing cognitive processing speed as measured by Symbol Digit Modalities Test (SDMT) (*d* = 0.9, *p* < 0.001) and verbal fluency (both category-based *d* = 0.87, *p* < 0.001; letter-based *d* = 0.67, *p* < 0.001). After adjusting for age, sex and years of education, MS/HC diagnosis was best predicted (*R*^2^ = 0.27) by differences in category-based verbal fluency (Wald = 9.935, *p* = 0.002) and SDMT (Wald = 13.937, *p* < 0.001).

**Conclusion:**

This study confirms the common hallmark of slowed cognitive processing speed in MS among elderly patients. Defective verbal fluency, less often observed in younger cohorts, may represent emerging cognitive pathology due to other etiologies.

## Introduction

Multiple sclerosis (MS) is a chronic demyelinating disease of the central nervous system (CNS) which presents with acute inflammatory episodes and co-occurring widespread neurodegeneration (Reich et al., 2018). These neuropathological processes are not only linked to physical disability progression, but also to cognitive impairment (Benedict et al., 2014). The increasingly favorable mortality prognosis with new disease modifying therapies raises new questions about quality of life, the influence of frailty, prevalence of comorbidities, and cognitive aging among the elderly with MS (EwMS) (Sanai et al., 2016; Koch-Henriksen et al., 2017).

Alzheimer’s disease (AD) and its prodromal state, amnestic mild cognitive impairment (aMCI), are among the many comorbidities expected to increase within the aging population. Furthermore, the progressive MS phase is associated with neurodegenerative features resembling an AD-like phenotype. Although both diseases may lead to dementia, their cognitive hallmarks are thought to differ. MS patients commonly present with slowed cognitive processing speed (CPS) and deficient learning, a cognitive profile frequently associated with lesion disruption of neural networks and atrophy of major hubs that facilitate information exchange (e.g., thalamus) (Van Schependom et al., 2015; Bergsland et al., 2018). Contrarily, AD is characterized by progressive decline in episodic memory, rapid forgetting, and impairments in semantic language. These defects are associated with neurodegeneration starting within the hippocampus, and entorhinal cortex (Hyman et al., 1984; McKhann et al., 2011; Reitz et al., 2011). The prevalence of MS in the United States is now estimated at one million persons, and the average age of the population is increasing (Nelson et al., 2019). Therefore, the possibility that an EwMS patient may develop AD should be considered with new onset cognitive complaints (Luczynski et al., 2018). Nevertheless, only a few case reports of MS and AD dual diagnosis are found in the literature (Luczynski et al., 2018). Therefore, understanding the cognitive profiles EwMS is highly warranted.

In a preliminary cross-sectional investigation, we compared EwMS (an average of 60 years old) with age and education-matched aMCI and AD patients (Roy et al., 2018). On a test of semantic verbal fluency, we found no significant difference between cognitively impaired MS and aMCI patients (Roy et al., 2018). Therefore, one may propose that as the MS cognitive decline progresses, additional cognitive features apart from processing speed are likely to emerge. Herein we endeavored to comprehensively examine the cognitive profile of a larger group of EwMS and age-matched healthy controls (HCs) using MS and AD-specific psychometric tests. We hypothesized that if EwMS have a true impairment in the language aspects of verbal fluency, this deficit should remain significant after controlling for the influence of cognitive processing speed.

## Materials and Methods

### Study Population

The study sample was composed of prospectively enrolled EwMS and HCs. The inclusion criteria for EwMS were (1) age ≥50 years old, (2) neuropsychological and clinical examination, and (3) MS diagnosis per 2010-revised McDonald criteria (Polman et al., 2011). The exclusion criteria for the EwMS consisted of (1) history of psychiatric and major depressive disorder prior to the onset of MS, (2) active relapse within 30 days of the examination, and (3) use of corticosteroids within 30 days of the study. Inclusion and exclusion criteria for HCs were (1) ≥50 years old, (2) neuropsychological examination, (3) self-reported intact cognition, (4) no history of neurological or medical illness that might impact cognitive function, and (5) no diagnosis of major depressive disorder. Furthermore, HCs with mini-mental state examination (MMSE) performance lower than 28 were excluded. Both the EwMS and HCs were additionally screened with the beck depression inventory-II fastscreen (BDI-FS).

Clinical examination was performed by a neurologist and physical disability was assessed using the expanded disability status scale (EDSS) (Kurtzke, 1983). The Timed 25-Foot Walk (T25FW) and 9-Hole Peg Test (9PHT) were additional measures of lower and upper extremity function, respectively. The EwMS were classified by disease course as relapsing-remitting MS (RRMS) and progressive MS (PMS). The study was approved by the Institutional Review Board (IRB) and all participants signed an informed consent form.

### Neuropsychological Examination

The neuropsychological examination was performed by trained examiners under supervision of a board-certified neuropsychologist (RHBB) and included tests traditionally regarded as MS and AD-specific (Strauss et al., 2006). The cognitive domains, their participating tests, and corresponding references are shown in Table 1.

**Table 1:** Cognitive domains and their respective tests.

Abbreviation	Name	References
**Processing speed/working memory**		
PASAT-3	Paced Auditory Serial Addition Test (3 second version)	Gronwall, 1977
SDMT	Symbol Digit Modalities Test	Smith, 1982
**Learning and memory**		
CVLT-II total learning trials	California Verbal Learning Test (total score)	Delis et al., 2000
CVLT-II short delay	California Verbal Learning Test (short delay score)	Delis et al., 2000
CVLT-II long delay	California Verbal Learning Test (long delay score)	Delis et al., 2000
CVLT-II recognition index	California Verbal Learning Test (recognition index)	Delis et al., 2000
BVMT-R total learning trials	Brief Visuospatial Memory Test – Revised (total immediate score)	Benedict, 1997
BVMT-R delay	Brief Visuospatial Memory Test – Revised (long delay score)	Benedict, 1997
BVMT-R recognition index	Brief Visuospatial Memory Test – Revised (recognition index)	Benedict, 1997
WMS-R Logical Memory IR	Wechsler Memory Scale – Revised (immediate recall score)	Wechsler, 1945
WMS-R Logical Memory DR	Wechsler Memory Scale – Revised (delayed recall score)	Wechsler, 1945
WMS-R Logical Memory MR	Wechsler Memory Scale – Revised (memory retention)	Wechsler, 1945
**Spatial processing**		
Beery VMI	Beery-Buktenica Visual-Motor Integration Test	Beery, 2010
Clock drawing test	N/A	Agrell and Dehlin, 1998
**Executive function**		
D-KEFS sorting CS	Delis-Kaplan Executive Function System (correct sorts score)	Delis et al., 2001
D-KEFS sorting DS	Delis-Kaplan Executive Function System (description score)	Delis et al., 2001
**Language**	
COWAT	Controlled Oral Word Association Test	Ruff et al., 1996
BNT	Boston Naming Test	Goodglass et al., 1983
Animal fluency	N/A	Ruff et al., 1996
Supermarket items fluency	N/A	Ruff et al., 1996
Total verbal fluency	N/A	Ruff et al., 1996


The exam included the Minimal Assessment of Cognitive Function in Multiple Sclerosis (MACFIMS) battery (Benedict et al., 2002) which evaluates several domains, including: processing speed/working memory [Paced Auditory Serial Addition Test (PASAT) (Gronwall, 1977) and Symbol Digit Modalities Test (SDMT) (Smith, 1982)], learning and memory [learning trials for California Verbal Learning Test–second edition (CVLT-II) (Delis et al., 2000) and Brief Visuospatial Memory Test-Revised (BVMT-R) (Benedict, 1997)], language [Controlled Oral Word Association Test (COWAT) (Ruff et al., 1996)] and executive function [number of correct sorts and description scores from the Sorting subtest of the Delis-Kaplan Executive Function System (D-KEFS) (Delis et al., 2001)].

Participants also completed neuropsychological measures that are traditionally utilized for detecting AD-specific cognitive impairments (Strauss et al., 2006). These tests included those measuring the domains of memory [Logical Memory from the Wechsler Memory Scale-Revised (WMS-R) (Wechsler, 1945)], language [Boston Naming Test (BNT) (Goodglass et al., 1983)], two categories of semantic fluency (total number of categorical items generated in one minute; animals and supermarket items), and visuospatial skill [Beery Visual-Motor Integration (VMI) (Beery, 2010) and clock drawing test (Agrell and Dehlin, 1998)]. The MMSE was also performed as an additional assessment of descriptive global cognitive functioning. For all performed psychometric tests, higher scores indicate better cognitive performance.

### Statistical Analysis

All statistical tests were performed using SPSS version 25.0 (IBM, Armonk, NY, United States). The distribution of the variables was examined using both Kolmogorov-Smirnov and Shapiro-Wilk tests of normality. The differences in demographics and cognitive raw scores between EwMS and HCs were determined by χ^2^–test for categorical variables, ANOVA or *t*-test for continuous, normally distributed variables, and Mann Whitney *U*-test for continuous, not normally distributed variables. To control for the multiple comparisons, Benjamini-Hochberg procedure was employed and adjusted *p* < 0.05 were considered statistically significant.

In order to delineate the degree of cognitive impairment across various cognitive domains while controlling for age, *z*-scores derived from the age-matched HCs were calculated. EwMS with *z*-score performance lower than -1.5 on at least two different cognitive domains were classified as cognitively impaired. The number of tests within each corresponding domain are shown in Table 1.

The impact of CPS on differences in cognitive performance between EwMS and HCs was additionally determined by analysis of covariance (ANCOVA) adjusted for SDMT scores.

Lastly, a multivariate logistic regression model determining the specific cognitive differences between the EwMS and the HCs was constructed. The model was composed of an initial force-entered block which corrected for differences in sex, age, and years of education. Furthermore, a second step-wise inclusion block determined the strength and order of cognitive variables which provided the greatest explanatory value in differentiating EwMS and HCs. For each derived step, the respective R^2^, Wald coefficient, and *p*-values were reported.

## Results

### Demographic and Clinical Characteristics

The EwMS (*n* = 104) and HCs (*n* = 56) were similar in age (62.1 vs. 62.3 years, *p* = 0.919), years of education (15.2 vs. 15.6 years, *p* = 0.213) and sex ratio (75.0% vs. 62.5% females, *p* = 0.09). The MS group had a mean disease duration of 20.5 years, median EDSS scores of 3.5 (IQR 3.0–6.0), and ratio of RRMS/PMS of 73/31. Regarding disease modifying therapy (DMT), most EwMS were prescribed interferon-β (32, 30.8%), followed by glatiramer acetate (28, 17.3%), oral DMT medications (15, 14.4%), natalizumab (6, 5.8%), off-label medication (1, 0.9%), and the rest of the EwMS were not on any DMT (32, 30.8%).

The EwMS were more disabled when compared to the HCs in both lower and upper extremity function (T25FW, 6.7s vs. 4.7s, *p* < 0.001; and 9PHT, 24.7s vs. 21.5s, *p* < 0.001). The MS group also had higher depression scores (BDI-FS of 1.0 vs. 0.0, *p* = 0.005). All demographic and clinical information are shown in Table 2.

**Table 2:** Demographic and clinical characteristics of the EwMS and HCs samples.

Demographic and clinical characteristics	EwMS (*n* = 104)	HCs (*n* = 56)	Cohen’s d	*p*-value
Female, n (%)	78 (75.0)	35 (62.5)	–	0.09
Age, mean (SD)	62.1 (6.3)	62.3 (8.1)	0.028	0.919
Years of education, mean (SD)	15.2 (2.3)	15.6 (2.3)	0.174	0.213
EDSS, median (IQR)	3.5 (3.0-6.0)	-	–	–
Disease duration, mean (SD)	20.5 (11.9)	-	–	–
RRMS/PMS	73/31	-	–	–
T25FW, median (IQR)	6.7 (5.1-9.1)	4.7 (4.1-5.1)	**0.514**	**<0.001**
9-PHT, median (IQR)	24.1 (22.1-29.5)	21.5 (20.4-23.5)	**0.532**	**<0.001**
BDI-II, median (IQR)	1.0 (0.0-2.75)	0.0 (0.0-2.0)	**0.374**	**0.027**
Disease modifying therapy, n (%)		
Interferon-β	32 (30.8)	-	–	–
Glatiramer acetate	18 (17.3)	-	–	–
Natalizumab	6 (5.8)	-	–	–
No DMT	32 (30.8)	-	–	–
Oral DMT^∗^	15 (14.4)	-	–	–
Off-label medications	1 (0.9)	-	–	–


*EwMS, elderly persons with multiple sclerosis; HCs, healthy controls; SD, standard deviation; IQR, interquartile ratio; RRMS, relapsing-remitting multiple sclerosis; PMS, progressive multiple sclerosis; BDI-II, Beck Depression Inventory; T25FW, Timed 25-Foot Walk; 9PHT, 9 Peg Hole Test; DMT, disease modifying therapy. Mann Whitney *U*-test, χ^*2*^, and Student’s *t*-test were used accordingly. *P*-value lower than 0.05 was considered statistically significant. ^∗^Oral DMTs include teriflunamide (9), dimethyl fumarate (5), and fingolimod (1). Use of off-label medications included only one case with rituximab. P-values lower than 0.05 were considered statistically significant and shown in bold.*

### Cognitive Performance, and *Z*-Score-Derived Domain-Specific Impairment Rates in the EwMS

The raw scores from the neuropsychological examination of the EwMS and HCs on both the MACFIMS and AD-specific batteries are shown in Table 3. EwMS had lower raw scores when compared to the age-matched controls on both total learned and short delay recall in CVLT-II (49.7 vs. 54.9, *d* = 0.45, *p* = 0.018 and 9.8 vs. 11.2, *d* = 0.38, *p* = 0.045, respectively), SDMT (46.3 vs. 55.4, *d* = 0.91, *p* < 0.001), letter verbal fluency (35.8 vs. 43.0, *d* = 0.67, *p* = 0.001) and category verbal fluency (17.3 vs. 20.3, *d* = 0.67, *p* = 0.001; 20.9 vs. 25.7, *d* = 0.84, *p* < 0.001; and 38.2 vs. 45.9, *d* = 0.87, *p* < 0.001, for animal category, supermarket category and total score, respectively). Furthermore, the EwMS had lower immediate recall and delayed recall scores on WMS-R logical memory performance (23.7 vs. 26.4, *d* = 0.39, *p* = 0.045 and 18.8 vs. 21.7, *d* = 0.38, *p* = 0.048). No other group differences were observed.

**Table 3:** Cognitive performance of the EwMS and HCs populations on the minimal assessment for cognitive function in multiple sclerosis (MACFIMS) battery.

Cognitive test	EwMS (*n* = 104)	HCs (*n* = 56)	Cohen’s d	*p*-value	BH-adjusted *p*-value^∗^
CVLT-II total learning trials	49.7 (12.5)	54.9 (10.4)	**0.452**	**0.005**	**0.018**
CVLT-II short delay	9.8 (4.1)	11.2 (3.3)	**0.376**	**0.02**	**0.045**
CVLT-II long delay	10.7 (3.9)	11.9 (3.3)	0.581	0.029	0.053
CVLT-II recognition index	2.9 (1.0)	3.3 (0.7)	0.463	0.111	0.167
BVMT-R total learning trials	19.9 (7.1)	22.5 (5.6)	0.407	0.018	0.05
BVMT-R delay	8.1 (2.7)	8.5 (2.7)	0.148	0.309	0.412
BVMT-R recognition index	5.5 (0.8)	5.5 (0.9)	0.0	0.634	0.667
SDMT	46.3 (10.8)	55.4 (9.2)	**0.907**	**<0.001**	**<0.001**
PASAT-3	41.5 (13.2)	45.8 (12.3)	0.337	0.05	0.077
D-KEFS sorting CS^a^	9.0 (2.8)	9.3 (2.2)	0.119	0.579	0.644
D-KEFS sorting DS^a^	33.6 (10.8)	34.9 (8.9)	0.131	0.448	0.559
BNT	56.1 (3.8)	56.9 (3.3)	0.225	0.179	0.256
Letter-based verbal fluency	35.8 (11.2)	43.0 (10.2)	**0.672**	**<0.001**	**<0.001**
Animal-based fluency	17.3 (4.7)	20.3 (4.3)	**0.666**	**<0.001**	**0.001**
Supermarket items-based fluency	20.9 (5.5)	25.7 (5.9)	**0.842**	**<0.001**	**<0.001**
Total categorical fluency	38.2 (8.8)	45.9 (8.9)	**0.870**	**<0.001**	**<0.001**
Beery VMI	23.2 (3.3)	24.2 (2.6)	0.337	0.03	0.05
Clock drawing test	8.9 (1.2)	9.1 (1.2)	0.167	0.505	0.594
WMS-R logical memory IR	23.7 (7.1)	26.4 (6.4)	**0.399**	**0.018**	**0.045**
WMS-R logical memory DR	18.8 (7.3)	21.7 (8.1)	**0.376**	**0.024**	**0.048**
WMS-R logical memory MR	78.1% (14.8%)	79.3% (18.3%)	0.072	0.641	0.641


*EwMS, elderly persons with multiple sclerosis; HC, healthy controls; MACFIMS, Minimal Assessment of Cognitive Function in Multiple Sclerosis; CVLT-II, California Verbal Learning Test; BVMT-R, Brief Visuospatial Memory Test – Revised; SDMT, Symbol Digit Modalities Test; PASAT-3, 3 second Paced Auditory Serial Addition Test; D-KEFS, Delis-Kaplan Executive Function System; CS, number of correct sorts; DS, description score; BNT, Boston Naming Test; VMI, Visual-Motor Integration; WMS-R, Wechsler Memory Scale – Revised; IR, immediate recall; DR, delayed recall; MR, memory retention. All variables are shown as the mean (SD) raw scores. For all tests, higher scores relate to better cognitive performance. Letter-based and categorical verbal fluency were examined by Controlled Oral Word Association Test (COWAT). Student’s *t*-test was used ^∗^Benjamini-Hochberg adjusted *p*-value <0.05 was considered statistically significant and shown in bold. *^*a*^*77 out of 104 EwMS patients had available D-KEFS scores.*

To investigate the influence of processing speed on our findings, we performed an analysis of covariance in which the COWAT differences (letter-based and category-based fluency) were adjusted by CPS performance (SDMT score as covariate). The performance on SDMT had a significant influence on letter-based verbal fluency performance (*F*_1,156_ = 39.5 *r* = 0.49, *p* < 0.001). In the final model, EwMS and HCs were not significantly different in letter-based fluency scores when CPS was controlled (*F*_1,156_ = 3.9, *p* = 0.057). Processing speed also influenced the performance on total categorical verbal fluency (*F*_1,156_ = 34.2, *r* = 0.425, *p* < 0.001) and on both animal-specific (*F*_1,156_ = 12.2, *r* = 0.242, *p* = 0.001), and supermarket items-specific category (*F*_1,156_ = 39.7, *r* = 0.476, *p* < 0.001). Despite the large SDMT effect, categorical verbal fluency remained significantly different between the EwMS and HCs (adjusted *p* = 0.036, adjusted *p* < 0.001, and adjusted *p* = 0.001, for animals, supermarket items, and total categorical verbal fluency, respectively).

Based on the HC-derived *z*-scores, the differences in processing speed and verbal fluency represented the most commonly affected domains in the EwMS. More than a quarter (27.9%) of EwMS had categorical verbal fluency impairment relative to HCs (22.1 and 24.0% for individual animal and supermarket tests, respectively). 25.0% were impaired on the letter-based verbal fluency, and 25.9% as impaired on the SDMT. Overall, 47.1% of the EwMS were classified as cognitively impaired on at least two different domain-specific tests. Table 4 portrays the impairment rate observed for each cognitive test.

**Table 4:** The ratio and percentage of cognitive impairment in the EwMS patients.

Psychometric test	Impairment ratio	Psychometric test	Impairment ratio
CVLT-II total learning trials	23/104 (22.1%)	D-KEFS Sorting DS	14/77 (13.5%)
CVLT-II short delay	23/104 (22.1%)	BNT	10/104 (9.6%)
CVLT-II long delay	18/104 (17.3%)	Animal-based fluency	23/104 (22.1%)
CVLT-II recognition index	21/104 (20.2%)	Supermarket items-based fluency	25/104 (24.0%)
BVMT-R total learning trials	23/104 (22.1%)	Total categorical fluency	29/104 (27.9%)
BVMT-R delay	11/104 (10.6%)	Beery VMI	23/104 (22.1%)
BVMT-R recognition index	12/104 (11.5%)	Clock drawing test	14/104 (13.5%)
SDMT	27/104 (25.9%)	WMS-R logical memory IR	19/104 (18.3%)
PASAT-3	18/104 (17.3%)	WMS-R logical memory DR	11/104 (10.6%)
Letter-based verbal fluency	26/104 (25.0%)	WMS-R logical memory MR	6/104 (5.8%)
D-KEFS Sorting CS	17/77 (16.3%)		


*CVLT-II, California Verbal Learning Test; SD, short delay; LD, long delay; BVMT-R, Brief Visuospatial Memory Test – Revised; SDMT, Symbol Digit Modalities Test; PASAT-3, 3 second Paced Auditory Serial Addition Test; COWAT, Controlled Oral Word Association Test; D-KEFS, Delis-Kaplan Executive Function System; CS, number of correct sorts; DS, description score; BNT, Boston Naming Test; VMI, Visual-Motor Integration; WMS-R, Wechsler Memory Scale – Revised; IR, immediate recall; DR, delayed recall; MR, memory retention. Cognitive impairment was defined as a score <-1.5 SD relative to mean HC performance on the respective test. Letter-based and categorical verbal fluency were examined by Controlled Oral Word Association Test (COWAT).*

Both the multivariate logistic regression model blocks and derived step-wise predictors are shown in Table 5. The diagnosis of MS was best predicted by differences in category-based verbal fluency (Wald = 9.935, *p* = 0.002) and SDMT (Wald = 13.937, *p* < 0.001). Together with the force-entered block containing age, sex and years of education, the final step-wise model was able to explain 27% of the diagnosis variance (Nagelkerke *R*^2^ = 0.27). In terms of odds ratio (OR), for every point decrease in categorical verbal fluency, the chance of MS diagnosis increased for 8.1% [Exp(B) = 0.919]. Similarly, for every point decrease in SDMT, the change of MS diagnosis is increased for 9.3% [Exp(B) = 0.907].

**Table 5:** Logistic regression model determining the cognitive differences between the EwMS and HCs.

	Nagelkerke R^2^	Wald coefficient	Exp(B)	*p*-value
**Block 1**	0.04			
Sex		3.186	1.914	0.074
Age		0.022	0.996	0.883
Years of education		1.973	0.901	0.16
**Block 2**				
Step 1:	0.218			
SDMT		22.992	0.885	**<0.001**
Step 2:	0.27			
SDMT		13.937	0.907	**<0.001**
Supermarket items-based fluency		9.935	0.919	**0.002**


*EwMS, elderly with multiple sclerosis; HCs, healthy controls; SDMT, Symbol Digit Modalities Test. Block 1 force-entered the demographic characteristics (sex, age and years of education) regardless if they were significantly associated with the diagnosis. A second step-wise block included only significant differentiators between the EwMS and HCs. P-values lower than 0.05 were considered statistically significant and shown in bold.*

Lastly, we investigated the associations between verbal fluency and cognitive processing speed with the overall disability scores as depicted by EDSS. Within the total sample of EwMS, EDSS scores were associated with both total verbal fluency (Spearman’s ranked correlation, *r*_s_ = -0.292, *p* = 0.003) and SDMT performance (*r*_s_ = -0.398, *p* < 0.001). Due to the nature of EDSS scoring system, we further delineated the EwMS into groups within the lower range of the score (EDSS scores between 0 and 4.0 which depend and reflect the neurological examination) and within the higher range of the score (EDSS scores ≥4.0 which solemnly depends on the walking ability) (van Munster and Uitdehaag, 2017; Jakimovski et al., 2018a). As expected, the associations between cognitive performance and EDSS scores were mainly present within the subset of EwMS with lower EDSS scores (total verbal fluency *r*_s_ = -0.341, *p* = 0.001 and SDMT *r*_s_ = -0.398, *p* < 0.001).

## Discussion

The main findings from this comprehensive neuropsychological evaluation of EwMS and age-matched HCs are grounded in two main themes. First, in addition to the previously established processing speed impairment, EwMS present with poorer verbal fluency (for both letter and category). Second, these findings were further extended by the logistic regression model which highlighted verbal fluency performance as second major differentiator between EwMS and HCs.

Currently there are few published analyses examining the question of comorbid aMCI/AD and cognitive aging in EwMS. Muller et al. (2013) used an AD–specific battery to examine 120 total age-, sex-, and education-matched SPMS, aMCI, and HCs and found word list recognition as the single metric distinguishing SPMS from aMCI – recognition memory was preserved in SPMS and defective in aMCI. Furthermore, they additionally showed that SPMS patients had similar performance on both letter-based and category-based verbal fluency when compared to the aMCI patients and significantly lower than HCs (Muller et al., 2013). In a similar study, EwMS were impaired on both processing speed and categorical fluency (Cohen’s *d* = 0.68) when compared to HCs (Roth et al., 2018). Therefore, both studies are conforming to the present results showing a high rate of processing speed impairment and diminished verbal fluency performance in EwMS, but no differences in the recognition indices of both verbal memory and visuospatial processing (Roy et al., 2018). Although language impairment is classically associated with an AD cognitive profile, the deficits in processing speed and verbal fluency were not accompanied by deficits in naming and memory consolidation. Therefore, the verbal fluency deficits found here are likely attributable to MS rather than co-morbid aMCI/AD.

Verbal fluency tasks allow the participants only 1 min to produce as many words that start with a certain letter of the alphabet or are part of a specific category (e.g., animals or supermarket items). Given this predetermined time limitation, verbal fluency performance may potentially be moderated by processing speed, as we found in our adjusted model. Report by Roth et al. (2018) showed that the EwMS have poorer categorical fluency when compared to HCs; however, after adjusting for CPS performance, these differences became non-significant. The discrepancy between our results and theirs is, however, minuscule (*d* 0.87 vs. 0.68 in Roth et al., 2018) – the partial correlation survived in our study with *p* = 0.036 with more statistical power by virtue of a larger sample. Devising new language tests that are less dependent on CPS would be of value in this area of investigation. Finally, determining the nature of the language impairment and its association with specific structural MS changes will allow better understanding of this particular cognitive ability. For example, while examining neurodegenerative MS patterns, studies demonstrated accelerated atrophy of the entorhinal cortex and the bilateral temporal lobes in a population of older (on average of 57 years old) SPMS patients (Steenwijk et al., 2016). These neurodegenerative changes located within AD-associated areas may provide explanation for the emerging semantic and logical memory deficit seen in our EwMS.

One limitation of this study is its cross-sectional research design. In order to better evaluate cognitive aging trajectories, ideally, one would evaluate these same cognitive functions over a period of three or more years, and include not only EwMS, but also aMCI patients. MRI-derived neurodegenerative outcome measures, amyloid PET imaging, assessment of known AD risk factors and the effect of other age-related comorbidities may further determine the nature of the semantic fluency impairment seen in EwMS (Jakimovski et al., 2018b). Lastly, global disability scores which are currently used in the MS field are not suitable in depicting the increasingly prevalent cognitive impairment within the aging MS population.

## Conclusion

In conclusion, roughly half of EwMS are impaired on tests of cognitive processing speed or verbal fluency. The deficit in verbal fluency is not a hallmark feature of MS associated cognitive disorder for the wider MS population, and may represent a unique quality in the EwMS neurocognitive profile. Further studies investigating the additional disease-related factors which influence cognitive aging in EwMS are warranted.

## Data Availability

The datasets generated for this study are available on request to the corresponding author.

## Ethics Statement

This study was carried out in accordance with the Institutional Review Board (IRB) with written informed consent from all subjects. All subjects gave written informed consent in accordance with the Declaration of Helsinki. The protocol was approved by the University at Buffalo Institutional Review Board.

## Author Contributions

RB and DJ conceived and designed the study. All authors analyzed and interpreted the data and critically revised the manuscript for important intellectual content. RB, RZ, and BW-G supervised the study.

## Conflict of Interest Statement

The authors declare that the research was conducted in the absence of any commercial or financial relationships that could be construed as a potential conflict of interest.

## References

[B1] Agrell B. Dehlin O. (1998). The clock-drawing test. *Age Ageing* 27 399–403. 10.1093/ageing/afs149 23144287

[B2] Beery K. (2010). *Beery VMI Administration, Scoring, and Teaching Manual.* Bloomington: Pearson.

[B3] Benedict R. H. (1997). *Brief Visuospatial Memory Test–Revised: Professional Manual.* Lutz, FL: PAR.

[B4] Benedict R. H. Fischer J. S. Archibald C. J. Arnett P. A. Beatty W. W. Bobholz J. (2002). Minimal neuropsychological assessment of MS patients: a consensus approach. *Clin. Neuropsychol.* 16 381–397. 10.1076/clin.16.3.381.13859 12607150

[B5] Benedict R. H. Morrow S. Rodgers J. Hojnacki D. Bucello M. A. Zivadinov R. (2014). Characterizing cognitive function during relapse in multiple sclerosis. *Mult. Scler.* 20 1745–1752. 10.1177/1352458514533229 24842959

[B6] Bergsland N. Schweser F. Dwyer M. G. Weinstock-Guttman B. Benedict R. H. B. Zivadinov R. (2018). Thalamic white matter in multiple sclerosis: a combined diffusion-tensor imaging and quantitative susceptibility mapping study. *Hum. Brain Mapp.* 39 4007–4017. 10.1002/hbm.24227 29923266 PMC6128742

[B7] Delis D. C. Kaplan E. Kramer J. H. (2001). *Delis-Kaplan Executive Function System^®^(D-KEFS^®^): Examiner’s Manual: Flexibility of Thinking, Concept Formation, Problem Solving, Planning, Creativity, Impluse Control, Inhibition.* Bloomington: Pearson.

[B8] Delis D. C. Kramer J. Kaplan E. Ober B. A. (2000). *CVLT-II: California Verbal Learning Test: Adult Version.* New York, NY: The Psychological Corporation.

[B9] Goodglass H. Kaplan E. Weintraub S. (1983). *Boston Naming Test.* Philadelphia, PA: Lea & Febiger.

[B10] Gronwall D. (1977). Paced auditory serial-addition task: a measure of recovery from concussion. *Percept. Motor Skills* 44 367–373. 10.2466/pms.1977.44.2.367 866038

[B11] Hyman B. T. Van Hoesen G. W. Damasio A. R. Barnes C. L. (1984). Alzheimer’s disease: cell-specific pathology isolates the hippocampal formation. *Science* 225 1168–1170. 10.1126/science.6474172 6474172

[B12] Jakimovski D. Benedict R. Marr K. Gandhi S. Bergsland N. Weinstock-Guttmam B. (2018a). Lower total cerebral arterial flow contributes to cognitive perofrmance in multiple sclerosis patients. *Mult. Scler.* 10.1177/1352458518819608 [Epub ahead of print]. 30625030 PMC6616024

[B13] Jakimovski D. Weinstock-Guttman B. Hagemeier J. Vaughn C. B. Kavak K. S. Gandhi S. (2018b). Walking disability measures in multiple sclerosis patients: correlations with MRI-derived global and microstructural damage. *J. Neurol. Sci.* 393 128–134. 10.1016/j.jns.2018.08.020 30165291

[B14] Koch-Henriksen N. Laursen B. Stenager E. Magyari M. (2017). Excess mortality among patients with multiple sclerosis in Denmark has dropped significantly over the past six decades: a population based study. *J. Neurol. Neurosurg. Psychiatry* 88 626–631. 10.1136/jnnp-2017-315907 28705951

[B15] Kurtzke J. F. (1983). Rating neurologic impairment in multiple sclerosis: an expanded disability status scale (EDSS). *Neurology* 33 1444–1452. 6685237 10.1212/wnl.33.11.1444

[B16] Luczynski P. Laule C. Hsiung G. R. Moore G. R. W. Tremlett H. (2018). Coexistence of multiple sclerosis and Alzheimer’s disease: a review. *Mult. Scler. Relat. Disord.* 27 232–238. 10.1016/j.msard.2018.10.109 30415025

[B17] McKhann G. M. Knopman D. S. Chertkow H. Hyman B. T. Jack C. R. Jr. Kawas C. H. (2011). The diagnosis of dementia due to Alzheimer’s disease: recommendations from the national institute on aging-Alzheimer’s association workgroups on diagnostic guidelines for Alzheimer’s disease. *Alzheimers Dement.* 7 263–269. 10.1016/j.jalz.2011.03.005 21514250 PMC3312024

[B18] Muller S. Saur R. Greve B. Melms A. Hautzinger M. Fallgatter A. J. (2013). Recognition performance differentiates between elderly patients in the long term course of secondary progressive multiple sclerosis and amnestic mild cognitive impairment. *Mult. Scler.* 19 799–805. 10.1177/1352458512461392 23166118

[B19] Nelson L. M. Wallin M. T. Marrie R. A. Culpepper W. J. Langer-Gould A. Campbell J. (2019). A new way to estimate neurologic disease prevalence in the United States: Illustrated with MS. *Neurology* 92 1–13. 10.1212/WNL.0000000000007044 30770422 PMC6442012

[B20] Polman C. H. Reingold S. C. Banwell B. Clanet M. Cohen J. A. Filippi M. (2011). Diagnostic criteria for multiple sclerosis: 2010 revisions to the McDonald criteria. *Ann. Neurol.* 69 292–302. 10.1002/ana.22366 21387374 PMC3084507

[B21] Reich D. S. Lucchinetti C. F. Calabresi P. A. (2018). Multiple sclerosis. *N. Engl. J. Med.* 378 169–180. 29320652 10.1056/NEJMra1401483 PMC6942519

[B22] Reitz C. Brayne C. Mayeux R. (2011). Epidemiology of Alzheimer disease. *Nat. Rev. Neurol.* 7 137–152. 10.1038/nrneurol.2011.2 21304480 PMC3339565

[B23] Roth A. K. Denney D. R. Burns J. M. Lynch S. G. (2018). Cognition in older patients with multiple sclerosis compared to patients with amnestic mild cognitive impairment and healthy older adults. *Neuropsychology* 32 654–663. 10.1037/neu0000453 29939057 PMC6126957

[B24] Roy S. Drake A. Snyder S. Cline B. Khan A. Fuchs T. (2018). Preliminary investigation of cognitive function in aged multiple sclerosis patients: challenges in detecting comorbid Alzheimer’s disease. *Mult. Scler. Relat. Disord.* 22 52–56. 10.1016/j.msard.2018.03.008 29574353

[B25] Ruff R. Light R. Parker S. Levin H. (1996). Benton controlled oral word association test: reliability and updated norms. *Arch. Clin. Neuropsychol.* 11 329–338. 10.1016/0887-6177(95)00033-x 14588937

[B26] Sanai S. A. Saini V. Benedict R. H. Zivadinov R. Teter B. E. Ramanathan M. (2016). Aging and multiple sclerosis. *Mult. Scler.* 22 717–725. 26895718 10.1177/1352458516634871

[B27] Smith A. (1982). *Symbol Digit Modalities Test.* Los Angeles, CA: Western Psychological Services.

[B28] Steenwijk M. D. Geurts J. J. Daams M. Tijms B. M. Wink A. M. Balk L. J. (2016). Cortical atrophy patterns in multiple sclerosis are non-random and clinically relevant. *Brain* 139 115–126. 10.1093/brain/awv337 26637488

[B29] Strauss E. Sherman E. Spreen O. (2006). *A Compendium of Neuropsychological Tests: Administration, Norms, and Commentary.* Oxford: Oxford University Press.

[B30] van Munster C. E. Uitdehaag B. M. (2017). Outcome measures in clinical trials for multiple sclerosis. *CNS Drugs* 31 217–236. 10.1007/s40263-017-0412-5 28185158 PMC5336539

[B31] Van Schependom J. D’hooghe M. B. Cleynhens K. D’hooge M. Haelewyck M. C. De Keyser J. (2015). Reduced information processing speed as primum movens for cognitive decline in MS. *Mult. Scler.* 21 83–91. 10.1177/1352458514537012 25013149

[B32] Wechsler D. (1945). *Wechsler Memory Scale.* Bloomington: Pearson.

